# Hepatic Angiosarcoma: A Challenging Diagnosis

**DOI:** 10.7759/cureus.3283

**Published:** 2018-09-11

**Authors:** Leon D Averbukh, Marianna G Mavilia, Michael M Einstein

**Affiliations:** 1 Internal Medicine, University of Connecticut Health Center, Hartford, USA; 2 Internal Medicine, University of Connecticut Health Center, Windsor, USA; 3 Medicine/Gastroenterology and Hepatology, Hartford Hospital, Hartford, USA

**Keywords:** hepatic angiosarcoma, hepatic malignancy, angiosarcoma

## Abstract

Hepatic angiosarcoma (HA) accounts for 2% of primary liver tumors. Though rare, it is exceptionally deadly. The initial presentation of HA is nonspecific and no tumor markers have been associated with it. In general, liver function is maintained until later stages of the disease, often leading to diagnosis once the disease is already advanced or metastatic. In this report, we present the case of a 54-year-old male whose vague symptoms and non-diagnostic liver biopsy delayed the diagnosis of a rapidly progressing HA.

## Introduction

Hepatic angiosarcoma (HA) is a particularly rare, non-cirrhotic, primary malignancy of the liver, accounting for 2% of liver cancers [1]. However, it is still considered to be the third most common primary hepatic malignancy. HA is a high-grade, aggressive tumor. It carries a grim prognosis, with an average life expectancy of 10 months, even with treatment including surgical resection. In 60% of patients, the malignancy is metastatic at the time of presentation and diagnosis [1].

Demographically, the malignancy has a male to female predominance of 3:1 with the majority of patients diagnosed in their sixth decade of life [1]. Though HA has been historically linked to environmental toxins, including thorotrast, arsenic, radiation, vinyl chloride, anabolic steroids, and exogenous estrogens, most cases of HA do not yield a causative factor [2]. In this report, we describe the case of a previously healthy male whose initial presentation of dyspnea lead to the diagnosis of HA.

## Case presentation

**Figure 1:**
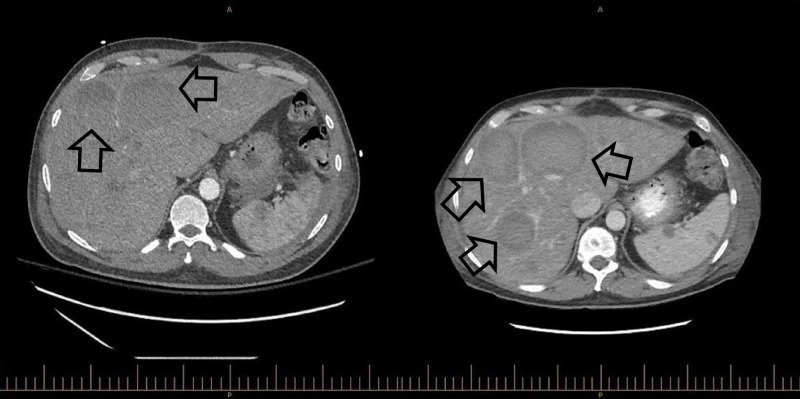
Side-by-side CT abdomen and pelvis with and without contrast imaging The study on the left was conducted at initial presentation and shows two liver lesions. The image on the right was performed one month later and demonstrated rapid disease progression with extensive metastatic disease infiltrating the parenchyma of the liver. CT: computed tomography

**Figure 2:**
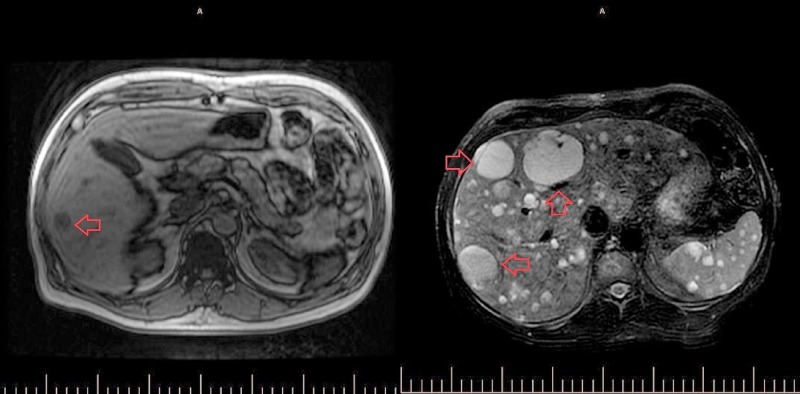
Side-by-side MRI abdomen and pelvis with and without contrast The study on the left was conducted at initial presentation and shows multiple liver lesions (small, dark gray lesions in the liver, the largest of which is highlighted with a red arrow). The follow-up MRI two months later revealed the progression of metastatic disease within the liver, spleen, and spine (light gray; the largest three lesions highlighted by the red arrows). MRI: magnetic resonance imaging; CT: computed tomography

The patient, a 54-year-old male with a past medical history significant for coronary artery disease, hypertension, and hyperlipidemia, initially presented with complaints of dyspnea on exertion for several weeks. The patient reported experiencing recent weight gain, increased abdominal girth, and lower extremity edema. An inpatient echocardiogram showed moderate pericardial effusion with possible markers for tamponade. He underwent a pericardial window computed tomography (CT) scan that showed two incidental hypoattenuating foci in the liver, the largest measuring 2.2 cm in diameter (Figure 1). There was no arterial enhancement within the lesions. Additional sub-centimeter hypo-attenuating foci were also noted but were too small to characterize by CT. A follow-up magnetic resonance imaging (MRI) scan of the abdomen and pelvis showed well-circumscribed T2 hyperintense lesions, which were hypo-enhancing to adjacent liver segments on post-contrast images (Figure 2). At the time of admission, the patient’s labs were as follows: total bilirubin 0.8 mg/dL, direct bilirubin 0.2 mg/dL, aspartate aminotransferase (AST) of 16 U/L, alanine aminotransferase (ALT) of 25 U/L, alkaline phosphatase (ALP) of 94 U/L, and platelet count of 177 Thou/uL. The patient later underwent an outpatient ultrasound-guided liver biopsy of the right lobe mass. Cytology did not reveal evidence of malignancy. Of note, the patient did not have a history of liver disease and denied any history of heavy alcohol use, drug use, exposure to viral hepatitis, or occupational exposures.

**Figure 3:**
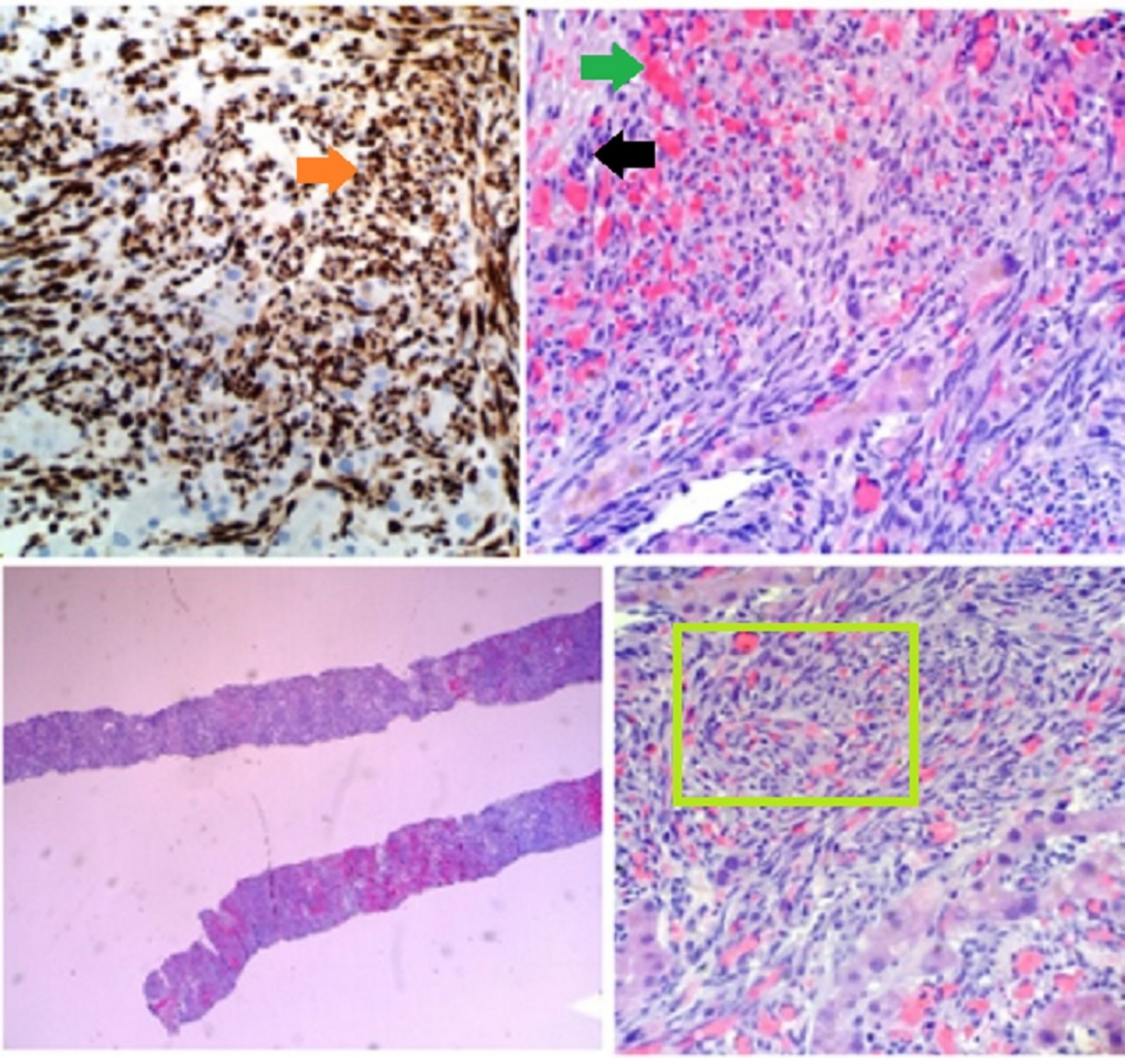
Hematoxylin and eosin stain preparations of the tumor from the second liver biopsy Demonstration of elongated spindle cells (black arrow) with vascular proliferation (green arrow) seen in the top-right and bottom-right (within green square) images. An example of the extent of malignant proliferation, leaving very few normal hepatic cells (bottom left). CD31 biomarker positive staining in brown seen in the top-left panel (orange arrow).

Two months later, the patient returned to the hospital due to increasing abdominal pain. A CT scan of the abdomen and pelvis showed new lesions and nodules as well as evidence of hemoperitoneum presumed to be due to ruptured hepatic and splenic lesions. At the time, his laboratory findings showed: total bilirubin 3.7 mg/Dl, direct bilirubin 1.0 mg/Dl, AST 108 U/L, ALT 105 U/L, ALP 250 U/L, platelet count 29 Thou/uL, and lactic acid 4.6 mmol/L. A second liver biopsy was performed and pathology showed solid spindle cell proliferation. Immunohistochemical staining was positive for cluster of differentiation (CD)31, CD34, and Factor VIII, indicating likely HA (Figure 3).

The patient was subsequently started on a cycle of gemcitabine. A follow-up MRI of the abdomen and pelvis two weeks later showed a progression of metastatic disease within the liver, spleen, spine, lung bases, and pericardium, with many of the metastases demonstrating signal characteristics consistent with interval hemorrhage (Figure 2). The largest lesion was seen in the left lobe of the liver, causing mass effect and left-sided intrahepatic biliary ductal dilatation. The patient experienced multiple complications of his disease, including hepatic encephalopathy, anasarca, septic shock, and right pseudo-atrial aneurysm. Regrettably, the patient expired seven months following his initial diagnosis of metastatic HA.

## Discussion

The liver makes up only 5% of all primary angiosarcoma lesion sites [3]. In cases of HA, the initial presentation is non-specific, including abdominal pain, weight loss, and fatigue. A physical exam is also of little diagnostic utility, as findings include hepatomegaly, ascites, and jaundice. As in our patient, spontaneous hemoperitoneum is common and highly suspicious for HA. Roughly half of the patients will have thrombocytopenia and elevated ALP [4]. There are no tumor markers that have been associated with HA and, in general, liver function is maintained until the late stages of the disease [4].

Radiologically, HAs vary in their appearance. Grossly, the tumor may appear with one of four different growth patterns: multinodular, a single dominant mass, mixed patterns of a dominant mass with smaller nodules, or an infiltrating micronodular tumor [2]. Metastases are common at the time of presentation, with the lungs, spleen, or bone being the most frequent secondary lesion sites. Unenhanced CT images of these masses are observed to be hypodense when compared to normal hepatic parenchyma while lesions observed with contrast-enhanced CT may be either hypo- or hyperdense, depending on the presence of hemorrhage within the tumor. Optimal imaging techniques for the diagnosis of HA include unenhanced, multiple-phase enhanced, and delayed CT or MRI to adequately capture all phases of the tumor and eliminate benign mimickers [5].

Primary HA is diagnosed based on the results of a histopathological examination. Though historically, HA has been linked with environmental toxins, including thorotrast, arsenic, radiation, vinyl chloride, anabolic steroids, and exogenous estrogens, most cases of HA never yield a causative factor. It should be noted that there is no difference in gross or microscopic pathology between the toxin-related HAs with their 20- to 30-year latency periods and idiopathic HAs [2]. On pathology, HAs range in appearance from epithelioid to spindled neoplasms, demonstrating various patterns of vascular channels [6]. In our patient’s case, some areas of hepatic parenchyma appeared to be completely replaced by solid spindle cell proliferation. Immuno-histological markers, which are often positive in HA, include CD31, CD34, erythroblast transformation-specific-related gene (ERG), Factor VIII, and vascular endothelial growth factor receptor-3 (VEGFR-3) [6]. Our patient’s parenchyma was positive for the markers CD31, CD34, and Factor VIII. Additionally, HAs present with tumor necrosis, which was also noted in our patient's case. What was unusual in our case was that the first biopsy of the tumor did not show signs of malignancy. Though there are no sensitivities reported on liver biopsy for HA, a literature review yielded only one case in which biopsy was non-contributory to disease identification over the span of five years until finally showing features of HA [1].

Treatment for HA has yielded generally poor outcomes due to the late stage and profound metastasis at diagnosis. Better prognosis is seen in patients that present with a single tumor mass, small tumor size, lack of metastases, low-grade lesion, and negative surgical resection margins [2]. For those without metastasis, surgical resection is the definitive treatment [3]. In the frequent cases of a multifocal or metastatic disease, adjuvant treatment remains the only choice, though no clear guidelines have been established on the optimal chemotherapeutic agents. Unfortunately, radiation therapy has had limited use, as it appears the cancer is radio-resistant [3]. Unlike what is seen in other liver conditions, such as alcoholic cirrhosis and primary biliary cirrhosis, a liver transplant is not favored as a treatment option, as tumor recurrence is high and long-term survival after transplant is, on average, only 23 months [7]. Our patient received chemotherapy with gemcitabine and, later, vinorelbine, as his multifocal and metastatic disease state made resection impossible. Unfortunately, the chemotherapy did not appear to significantly alter his disease course.

## Conclusions

HA is a malignancy that combines a nonspecific presentation with a very rapid progression of illness and, ultimately, death. While imaging and biopsy are important instruments in disease diagnosis, their sensitivities are not absolute. Early clinical suspicion and surgical intervention remain paramount in reducing the risk of mortality in HA.
